# Laticifer growth pattern is guided by cytoskeleton organization

**DOI:** 10.3389/fpls.2022.971235

**Published:** 2022-09-28

**Authors:** Maria Camila Medina, Mariane S. Sousa-Baena, Marie-Anne Van Sluys, Diego Demarco

**Affiliations:** Departamento de Botânica, Instituto de Biociências, Universidade de São Paulo, São Paulo, SP, Brazil

**Keywords:** laticifers, cytoskeleton, microtubules, development, apical growth

## Abstract

Laticifers are secretory structures that produce latex, forming a specialized defense system against herbivory. Studies using anatomical approaches to investigate laticifer growth patterns have described their origin; however, their mode of growth, i.e., whether growth is intrusive or diffuse, remains unclear. Studies investigating how cytoskeleton filaments may influence laticifer shape establishment and growth patterns are lacking. In this study, we combined microtubule immunostaining and developmental anatomy to investigate the growth patterns in different types of laticifers. Standard anatomical methods were used to study laticifer development. Microtubules were labelled through immunolocalization of α-tubulin in three types of laticifers from three different plant species: nonanastomosing (*Urvillea ulmacea*), anastomosing unbranched with partial degradation of terminal cell walls (*Ipomoea nil*), and anastomosing branched laticifers with early and complete degradation of terminal cell walls (*Asclepias curassavica*). In both nonanastomosing and anastomosing laticifers, as well as in differentiating meristematic cells, parenchyma cells and idioblasts, microtubules were perpendicularly aligned to the cell growth axis. The analyses of laticifer microtubule orientation revealed an arrangement that corresponds to those cells that grow diffusely within the plant body. Nonanastomosing and anastomosing laticifers, branched or not, have a pattern which indicates diffuse growth. This innovative study on secretory structures represents a major advance in the knowledge of laticifers and their growth mode.

## Introduction

Laticifers are formed by specialized cells which contain latex and form a defense system, sealing wounds, blocking microorganisms, and avoiding herbivory (Fahn, 1988; Agrawal and Fishbein, 2006; Demarco et al., 2013; Ramos et al., 2019, 2020). Laticifers can be a single cell (nonarticulated) or a row of cells (articulated). In the latter, the terminal walls of each cell in the row may remain intact (nonanastomosing) or can be partially or completely (anastomosing) dissolved (Fahn, 1979). In some cases, anastomosing laticifers can also branch through lateral anastomosis between two laticifer rows, forming an interconnected system of tubes throughout the entire plant (Demarco et al., 2006; Ramos et al., 2019; Johnson et al., 2021). Although these general aspects of laticifer development are well-known, how the laticifers grow is a question that is still postulated (Gama et al., 2017; Johnson et al., 2021).

Two concurrent hypotheses based on observations from anatomical and developmental studies have been debated to explain their mode of growth in the plant body: (1) Laticifers grow through apical intrusive growth in meristematic regions, dissolving the middle lamella through enzymatic activity and growing intrusively between cells (Mahlberg, 1993; Canaveze and Machado, 2016; Castelblanque et al., 2016; Canaveze et al., 2019). In this case laticifers might combine two types of cell expansion, diffuse growth followed by polarized growth, as described for some fibers (Snegireva et al., 2010; Gorshkova et al., 2012; Majda et al., 2021); (2) Laticifers may grow through the addition of new cells in the laticifer system followed by cell expansion, thus not through apical growth. Evidence supporting this view is the fact that laticifer apices (the region of the laticifer system where new cells that have just differentiated are added to the system) are found close to the shoot apical meristem but never penetrate this tissue (Milanez, 1960, 1977, 1978; Demarco and Castro, 2008; Demarco et al., 2013; Gama et al., 2017; Ramos et al., 2019; Naidoo et al., 2020).

Plant cells can grow diffusely or apically. The first process is typically anisotropic and occurs in almost all cells (Hamada, 2014). The second one is considered an extreme form of cell growth that is concentrated at the apex of the cell, which gives it the ability to grow extensively in a polarized way (Anderhag et al., 2000; Wasteneys and Galway, 2003). Apical growth has been associated with the search for nutrients, as observed in root hairs (Hepler et al., 2001; Sieberer et al., 2005) and also in pollen tubes searching for the ovule (Cai et al., 2017). Both root hairs and pollen tubes are models of apical growth for which important processes involving the cytoskeleton are well-characterized (Gu and Nielsen, 2013). Additionally, some plant cells display an apical intrusive growth where the plant cell grows differentially at its tip. Intrusive growth implies that a part of the cell maintains cell-to-cell contact with their neighboring cells, and another part of the cell (the tip) invades new locations (Lev-Yadun, 2001), provoking changes not only in cytoskeleton patterns but also in the middle lamella of the surrounding cells, as well as disrupting their plasmodesmata connections (Lev-Yadun, 2001; Majda et al., 2021; Box 1). The apical intrusive growth has also been used to explain the way laticifers might grow within the plant, i.e., combining an initial phase of diffuse growth and then a phase of apical growth during their development, as is observed in vascular fibers (Ageeva et al., 2005; Snegireva et al., 2010; Gorshkova et al., 2018; Gorshkov et al., 2019; Majda et al., 2021; Sousa-Baena and Onyenedum, 2022).

**BOX 1. Defining terms of plant cell growth modes**.

**Polarized growth:** It is a mode of growth in which cells expand in a unidirectional way due to an asymmetrical distribution of molecules and structures at the subcellular level. Different types and levels of polarity exist even in the same plant cell. Polarized growth occurs in plant cells with diffuse growth (anisotropic) and with tip growth.

**Diffuse growth:** This is the most common mode of growth in plant cells in which wall extension and incorporation of new wall material occurs uniformly across the cell surface. It can be isotropic (considered transitory in plant cell growth) or anisotropic.

**Apical or tip growth:** This is an extremely polarized mode of cell growth where wall synthesis occurs at a single site on the cell surface (e.g., pollen tubes or root hairs).

**Intrusive growth:** The plant cell has an apical growth that can be unidirectional or bidirectional, and the region that is differentially growing loses the cell-to-cell contact and invades intercellular spaces, damaging the middle lamella (e.g., some xylem fibers, phloem fibers, and gelatinous fibers).

The main characteristic of apical growth is the expansion of a single region of the wall independently of the rest of cell. This extremely polarized type of growth is mediated and directed by the cytoskeleton (Wasteneys and Galway, 2003; Smith and Oppenheimer, 2005; Li et al., 2017). Cells with apical growth usually have microtubules in longitudinal or slightly helical organization in the cortical and endoplasmic cell regions. Apparently, this organization facilitates the transport of secretory vesicles containing mainly pectin, which is deposited in the apical cell wall region (Anderhag et al., 2000). This local increment of wall constituents enlarges the cell, which maintains its cylindrical shape (Hepler et al., 2001), an essential feature for the preservation of apical growth both in roots and in pollen tubes (Gu and Nielsen, 2013).

Laticifer development has been widely studied using anatomical approaches. However, laticifers may grow in a diverse mode, with ramifications and turns inside the plant body, which is difficult to follow and interpret using exclusively structural ontogenetic methodologies. The observation of an acute apex in some laticifers has frequently been interpreted as evidence that they have apical growth; however, the acute shape of the cell tip in the apical region of the laticifer may, in fact, be the result of an oblique section of the sinuous apical portion of the laticifers (Gama et al., 2017). Therefore, new approaches are required to assess this question.

It is well known that cytoskeleton plays a crucial role in plant cell developmental processes and in the establishment of polarized growth (Kost et al., 1999), but no cytoskeleton analyses have been made in laticifers so far. Thus, we performed a comparative study of the role of microtubules in the growth mode of different types of laticifers in three species: (1) *Asclepias curassavica* L. (Apocynaceae), whose laticifers are anastomosing branched with early and complete degradation of terminal walls (Demarco and Castro, 2008), (2) *Ipomoea nil* (L.) Roth (Convolvulaceae), which has anastomosing unbranched laticifers with a partial degradation of terminal walls (pers. obs.) and (3) *Urvillea ulmacea* Kunth (Sapindaceae) with nonanastomosing laticifers (Medina et al., 2021).

## Materials and methods

### Sampling and cultivation

Shoots of *Asclepias* *curassavica* L. (Apocynaceae) and *Urvillea ulmacea* Kunth (Sapindaceae) were collected on the campus of the Universidade de São Paulo (USP) in São Paulo/SP (Brazil); vouchers for these specimens were deposited in the Herbarium SPF (SPF 150070 and SPF 227683, respectively). Shoots from *Ipomoea nil* (L.) Roth (Convolvulaceae) were collected from plants cultivated in the greenhouse from seeds acquired from Cosmos Agrícola Produção e Serviços Rurais Ltda. (Engenheiro Coelho/SP, Brazil).

### Developmental analysis

Shoot apices of *Asclepias*, *Ipomoea* and *Urvillea* were isolated and fixed in formalin-acetic acid-50% alcohol (FAA) for 24 h (Johansen, 1940) and stored in 70% ethanol. Then, the material was dehydrated in an ascending butyl series (Johansen, 1940) and embedded in Paraplast (Leica Microsystems, Heidelberg, Germany). All samples were longitudinally sectioned using a Microm HM340E rotary microtome (Microm, Walldorf, Germany) and then stained with 1% astra blue and 1% safranin (Gerlach, 1984). Slides were mounted in Permount resin (Fisher Scientific, Pittsburgh, PA, United States) and photographed using a Leica DMLB light microscope.

### Microtubules immunolocalization

A modified protocol based on Wasteneys et al. (1997) and Celler et al. (2016) was used for microtubule labelling. Peelings of the shoot apices of the three species, containing epidermis plus some stem cortical layers, were gently collected with forceps and then fixed in PMET buffer (100 mM PIPES, 5 mM EGTA, 1 mM magnesium sulfate, pH 6.9) containing 0.5% glutaraldehyde, 1.5% formaldehyde fixative solution for 40 min. Then, the peels were washed in PMET buffer (100 mM PIPES, 5 mM EGTA, 1 mM magnesium sulfate, 0.05% triton X-100, pH 6.9). A freeze-shattering procedure was performed following Wasteneys et al. (1997). An enzymatic cell wall digestion step was subsequently done using a 0.1% pectinase solution (0.1% pectinase from *Aspergillus aculeatus* Sigma-Aldrich, 0.4 M mannitol, 1% BSA and 1xPBS) for 20 min at room temperature. After rinsing off the pectinase solution with PMET buffer, samples were incubated for 3 h in the permeabilization buffer (PBS, 1% Triton X-100 in 1x PBS, pH 7.5) at room temperature. After washing in PBS, samples were incubated in a sodium borohydride solution (1 mg/mL sodium borohydride in 1x PBS) and then transferred to blocking buffer (1% BSA, 50 mM glycine in 1 x PBS).

All samples were incubated in a 1.5 mL Eppendorf tube with a solution 1:1000 of the monoclonal mouse anti-α-tubulin antibody, clone B 512 (Sigma-Aldrich catalogue number T6199) in blocking buffer at 4°C overnight. Five washes of 10 min each in the incubation buffer (50 mM glycine in 1x PBS) were carried out, and then the samples were transferred to the blocking buffer for 30 min. After that, the samples were incubated in a 1.5 mL Eppendorf tube with the secondary antibody in a 1:100 solution of Alexa 488-conjugated goat anti-mouse IgG antibody (Invitrogen catalogue number A28175) in blocking buffer for 3 h at 37°C. Samples were washed in PBS and mounted in antifade mounting medium (glycerin in PBS). The slides were analyzed using a Zeiss LSM880 confocal laser microscope at 488 nm wavelength. To observe the organization of microtubules in meristematic regions during development, z-stack images were created.

## Results

### Laticifer characterization and development

#### Branched laticifers

**Figure 1:**
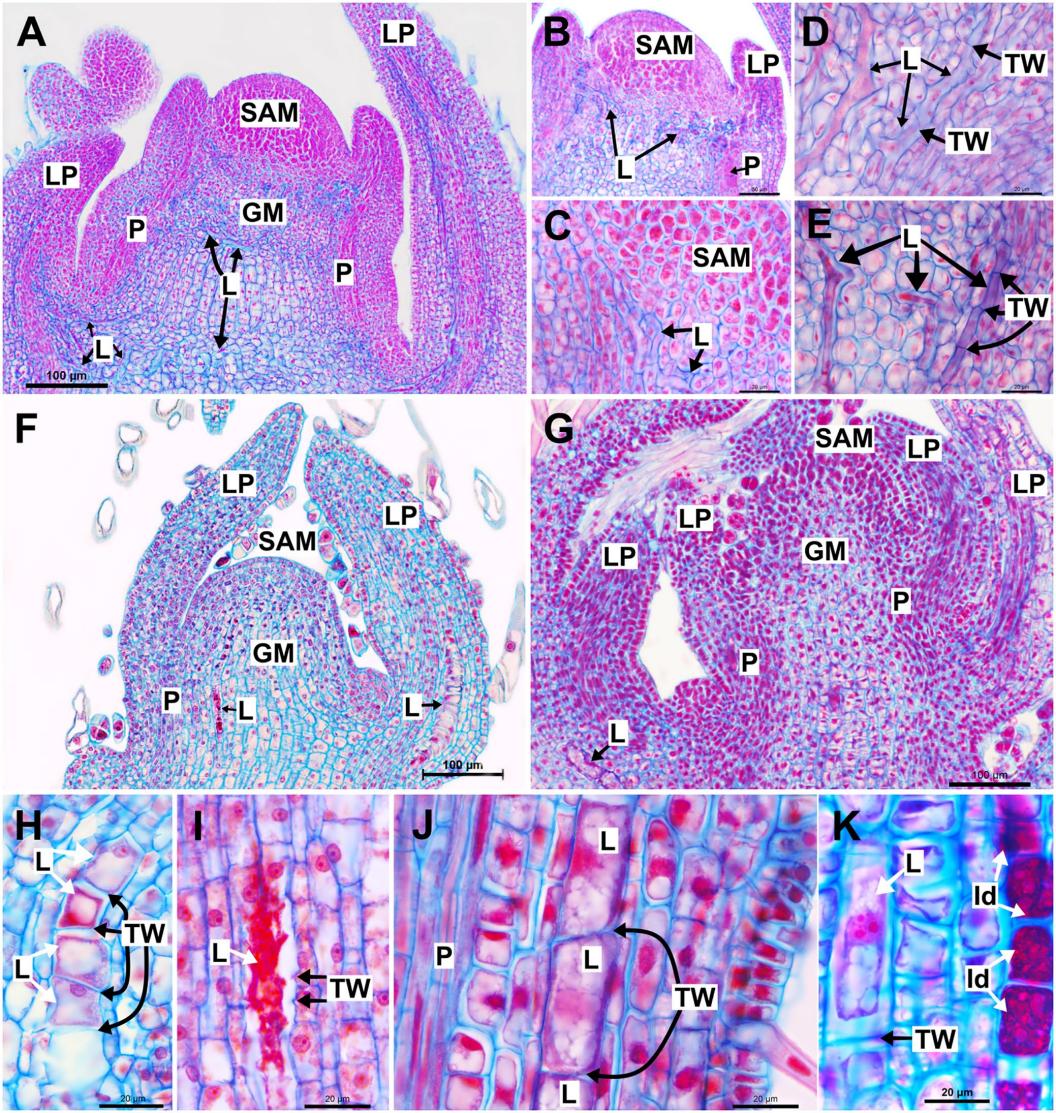
Structure and development of laticifers in *Asclepias curassavica*, *Ipomoea nil* and *Urvillea ulmacea*. **(A–E)** Anastomosing branched laticifers of *A. curassavica*. **(F,H,I)** Anastomosing unbranched laticifers of *I. nil*. **(G,J,K)** Nonanastomosing unbranched laticifers of *U. ulmacea*. (GM, ground meristem; Id: idioblast; L, laticifer; LP, leaf primordia; N, nucleus; P, procambium; SAM, shoot apical meristem; TW, terminal wall).

In the shoot system of *Asclepias curassavica*, whose laticifers are anastomosing branched with early and complete degradation of terminal walls, laticifers are found in the stem cortex, vascular system and pith and in the mesophyll and vascular system of leaves (Figure 1A). They are originated from the ground meristem and procambium, and their apices are located near the shoot apical meristem (SAM) and within leaf primordia (Figures 1B,C). Each laticifer is formed by a row of cells and develop in meristematic regions, where intercellular spaces are absent. The apical cells of the laticifer differentiate rapidly, and their walls are thicker than the adjacent cells (Figures 1D,E). The dissolution of the terminal walls (transverse walls located between two laticifer cells) within the laticifer occurs early in development, and only a few cells with entire terminal walls are observed in apical portions of the shoot. Even in portions very close to the SAM, the terminal walls of some laticifers were already dissolved, with the laticifer forming a continuous tube-like structure (Figures 1B–E). In the same way, the production of latex, as well as laticifer branching, begins early, still in the meristematic region of the stem (Figure 1E).

#### Unbranched laticifers

Similar to laticifers of *Asclepias*, laticifers of *Ipomoea*, which are anastomosing unbranched with a partial degradation of terminal walls, and *Urvillea*, which are nonanastomosing, are formed and grow in the shoot apices, but in such species, they are originated only from the ground meristem in the cortex and pith (Figures 1F,G). They are formed by a row of cells, more or less straight, and can be distinguished from neighboring cells by latex content which is precociously produced (Figure 1H). Laticifers of *Ipomoea* are anastomosed with partial, late disintegration of the terminal walls, whose debris can be observed in mature portions of the laticifer (Figure 1I). Conversely, laticifers of *Urvillea* are nonanastomosing, maintaining the terminal walls intact throughout development (Figure 1J). It is noteworthy that the laticifers of Ipomoea and *Urvillea* are much larger in expanding leaf primordia when compared to those of *Asclepias* (Figures 1F,G). Unlike *Asclepias* and *Ipomoea*, *Urvillea* also have idioblasts in the shoot system, a secretory structure constituted by a single cell that produces mainly phenolic compounds (Figure 1K).

### Laticifer microtubules organization

**Figure 2:**
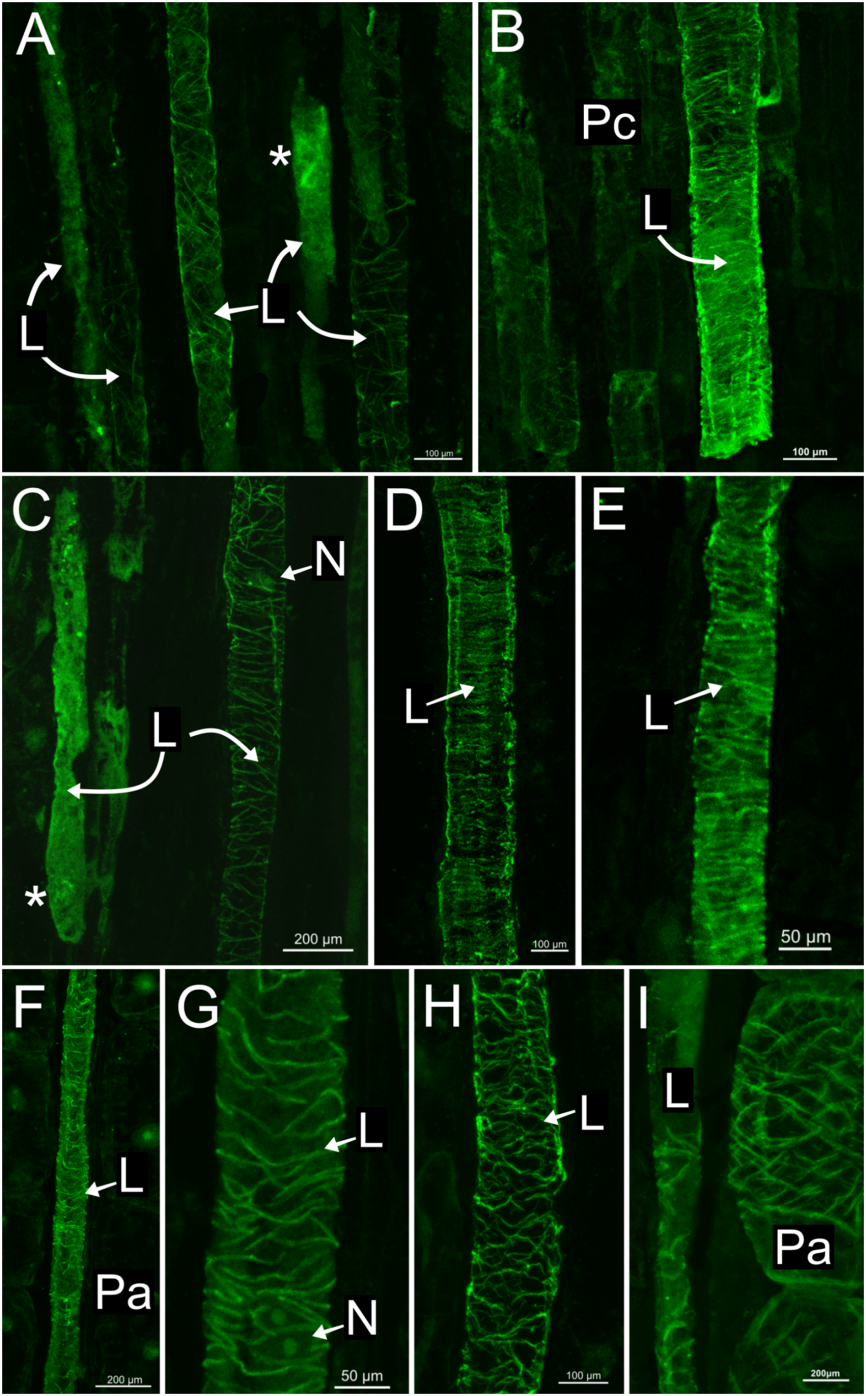
Immunolocalization of microtubules in articulated anastomosing laticifers of the shoot apex of *Asclepias* curassavica. **(A)** Procambial laticifers with cortical microtubules in transversal orientation to the cell axis. **(B,C,H,I)** Procambial laticifers showing a well-preserved transverse cortical microtubule orientation. Note the nucleus and a laticifer full of latex without microtubule detection in C. **(D,E)** Laticifers in the pith of stem exhibiting similar transverse microtubule arrangement. **(F,G)** Laticifer in cortex region of stem with some cortical microtubules. Note the nucleus in G. **(I)** Parenchyma cell with a transverse microtubule arrangement similar to the adjacent laticifer (asterisk, endoplasmic microtubules; L, laticifer; N, nucleus; Pa, parenchyma cell; Pc, procambium).

**Figure 3:**
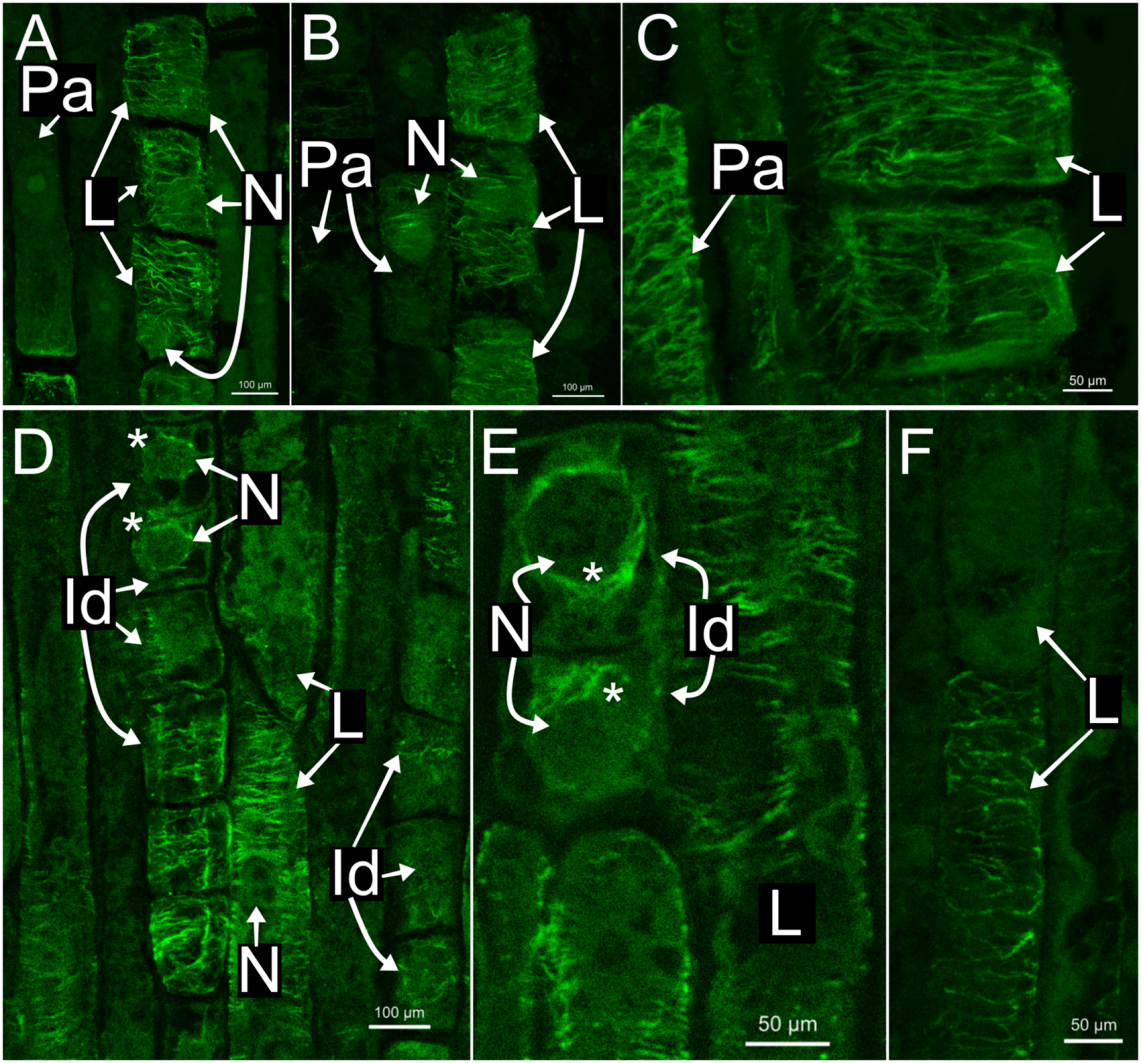
Immunolocalization of microtubules in articulated anastomosing laticifers of the shoot apices of *Ipomoea nil* **(A–C)** and *Urvillea ulmacea* **(D–F)**. Laticifers with transverse microtubule orientation in both species. **(A,B)** Laticifer nuclei are displaced to a parietal position due to the enlargement of the vacuole. **(C–E)** Both parenchyma cells and idioblasts with transverse microtubule arrangement similar to the adjacent laticifers. **(D,E)** Endoplasmic microtubules (asterisk) surrounding the nuclei of idioblasts (Id, idioblast; L, laticifer; N, nucleus; Pa, parenchyma cell).

**Figure 4:**
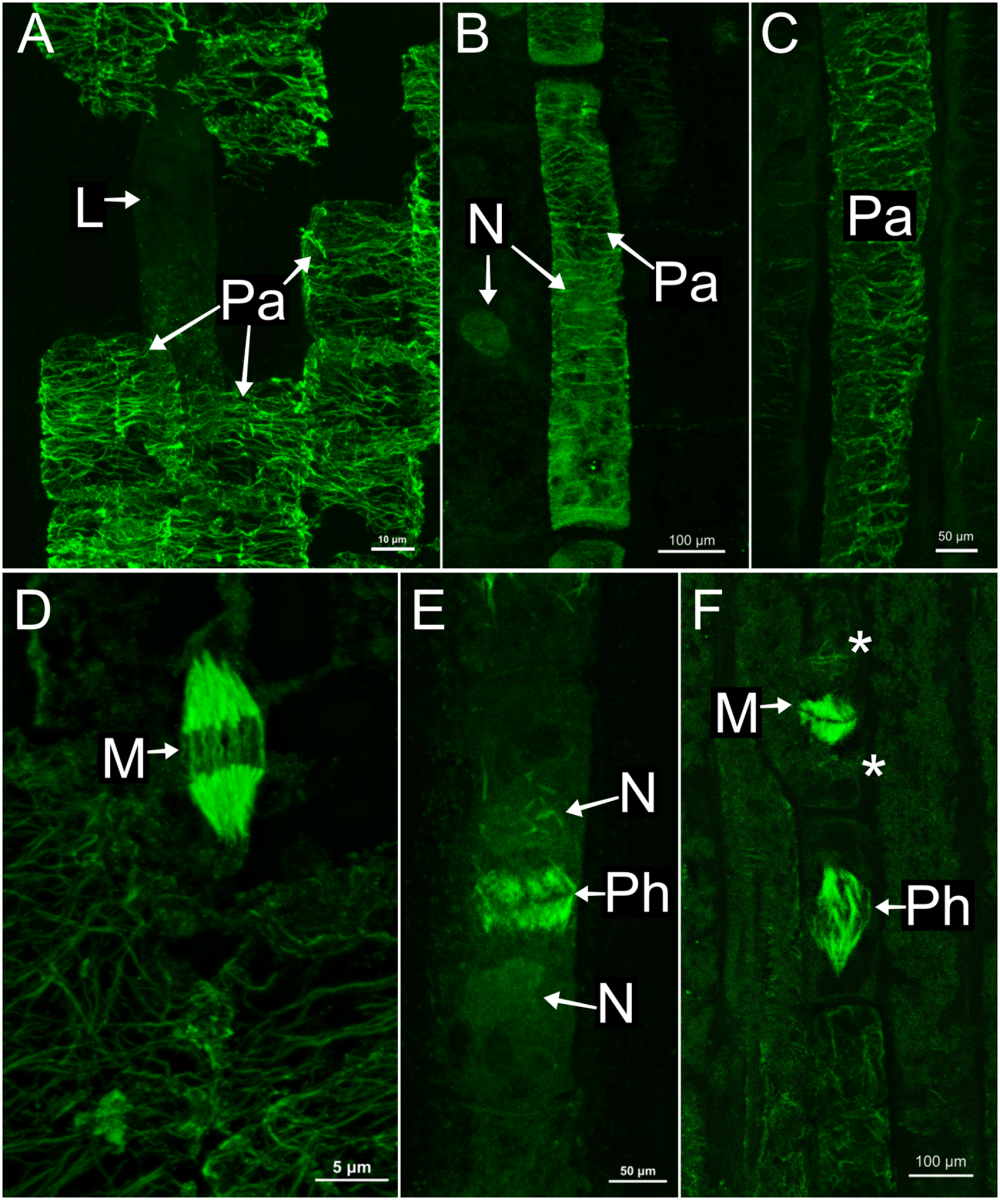
Immunolocalization of microtubules in parenchyma cells of the shoot apices. **(A,D)** *Asclepias* curassavica. **(B,E)** *Ipomoea nil*. **(C,F)** *Urvillea ulmacea*. **(A–C)** Cortical microtubules arranged perpendicularly to the cell axis. **(D–F)** Mitotic activity in meristematic regions. **(D,F)** Microtubules of the mitotic spindles. **(E)** Microtubules of the phragmoplast. (L, laticifer; M, mitosis; N, nucleus; Pa, parenchyma cell; Ph, phragmoplast).

**Figure 5:**
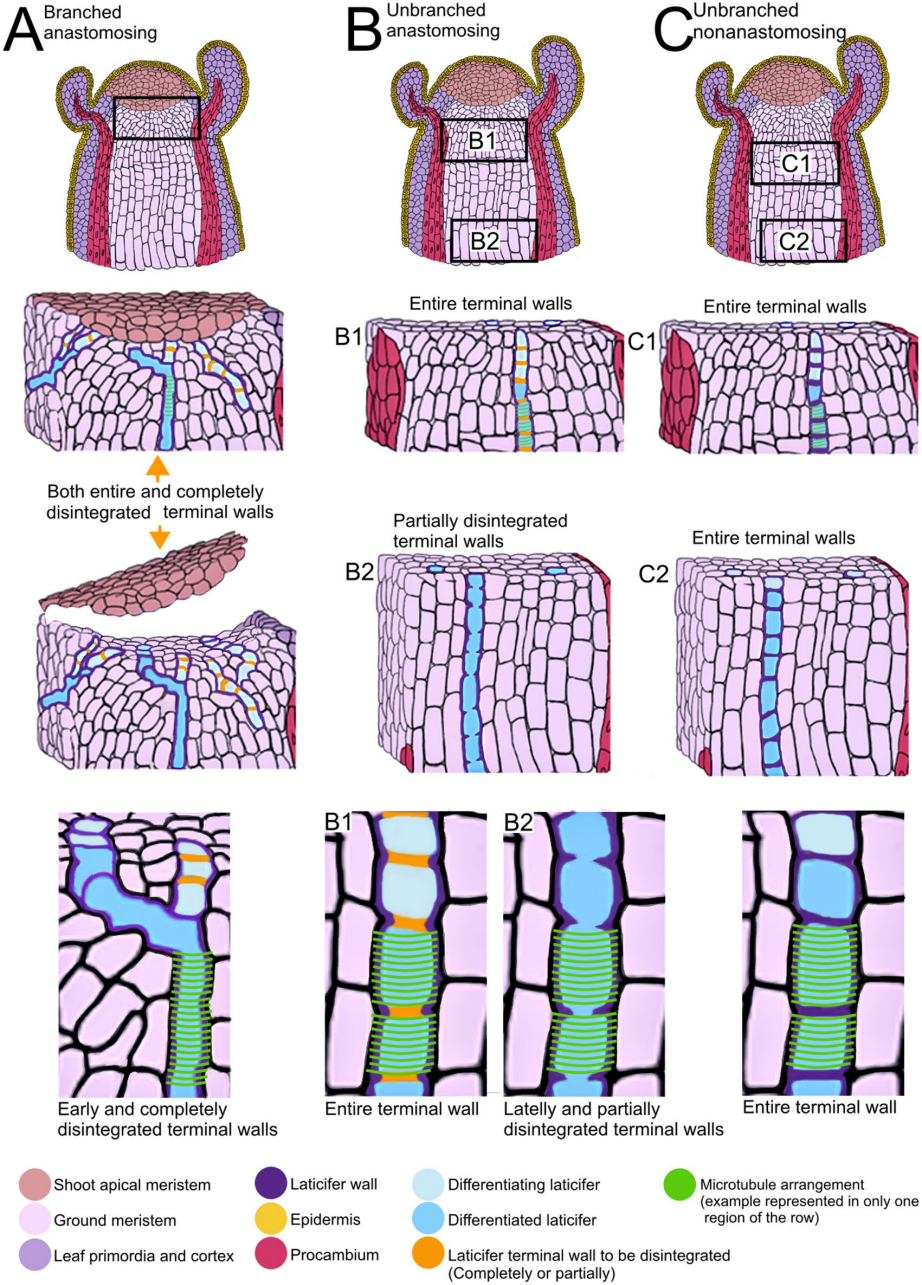
Summary of developmental processes of articulated laticifers and their microtubule patterns. **(A)** Articulated anastomosing branched laticifers. These laticifers differentiate early in development, branching and dissolving their terminal walls near the shoot apical meristem. **(B)** Articulated anastomosing unbranched laticifers. This type of laticifer forms more or less straight rows of cells, whose terminal walls may remain entire in meristematic regions (B1) but partially disintegrate lately, forming a continuous tube (B2). **(C)** Articulated nonanastomosing unbranched laticifers. This type of laticifer is differentiated in regions a little further from the shoot apical meristem (C1) and forms more or less straight rows of cells, whose terminal walls remain entire (C2).

In all species, developing and mature laticifers have cortical microtubules arranged perpendicular to the cell growth axis (Figures 2–5). A dense network of endoplasmic microtubules was also observed in developing laticifers of *Asclepias* (Figures 2A,C) idioblasts of *Urvillea* (Figures 3D,E) and meristematic cells (Figure 4F). Perpendicularly-oriented cortical microtubules were also observed in idioblasts cells (Figure 3D), parenchyma (Figures 4A–C) and ground meristem (Figures 4D,F).

Nuclei were evident in developing laticifers of all species (Figures 2G, 3A,B,D). Nuclei are parietally located due to the displacement caused by the expanding vacuole as observed in laticifers of *Ipomoea* (Figure 3A) and centrally located as in laticifers and idioblasts in *U. ulmacea* and parenchyma (Figures 3D,E). In laticifers, the displacement of nuclei to a parietal position is due to the production and accumulation of latex constituents within the vacuole, which compresses the nucleus against the wall. Microtubules were also observed as mitotic spindles and phragmoplasts in many meristematic cells in all species (Figures 4D–F).

See Figure 5 for a summary of the type of development of unbranched and branched laticifers and their pattern of microtubule arrangement.

## Discussion

In this study we show that cortical microtubules have the same arrangement, i.e., being organized perpendicular to the cell growth axis, in the three types of laticifers present in the different species analyzed in this study, as well as in the meristematic cells of ground meristem, in subepidermal parenchyma cells and phenolic idioblasts. Although both diffuse and polarized growth have been associated with laticifers, we found that cortical microtubules in nonanastomosing and anastomosing laticifers exhibited a transverse arrangement, commonly associated with diffuse growth, as opposed to a longitudinal organization, historically associated with apical and intrusive growth in other cell types, which strongly suggest that laticifers grow mainly through diffuse growth.

### Laticifers maintain adhesion to neighboring cell walls throughout development

Our analyses show that laticifers grow synchronously with other meristematic cells in the meristematic regions, as the middle lamella keeps the cells united across the whole process. Hence, the surface contact area increases at a similar rate, even when some cells assume different positions and shapes. This type of growth was described as symplastic growth by Fahn (1990). In particular, we observed that nonanastomosing laticifers can be longer and wider than the neighboring cells, which is probably related with differences in turgor pressure in the laticifer cells, which have a unique and distinct metabolism, and with their cell wall composition, as laticifer walls can be more acidic and thicker than neighboring cells in meristematic regions and also exhibit singular mechanical properties (Demarco et al., 2006; Palin and Geitmann, 2012; Demarco, 2015; Medina et al., 2021).

The apical intrusive growth of internal cells might require neighboring cells forming intercellular spaces by detachment of middle lamella in order to intrusively grow between the spaces formed. In that case, the region of the cell which is intrusively growing detaches from the neighboring walls. That process was observed, for instance, during xylary fiber tip growth (Majda et al., 2021). In phloem bast fibers, a higher rate of elongation in comparison with the neighboring cells has been also observed. In early stages of development, bast fibers have a symplastic growth from the region right below the SAM to a portion of the stem called “snap point” in flax (also observed in hemp and ramie; Sousa-Baena and Onyenedum, 2022); they start to grow in a polarized manner, displaying an intrusive growth with synchronous nuclear divisions. In addition, the transcriptome profile of isolated phloem bast fibers during their intrusive stage corroborate their intrusive nature as many genes, transcripts and miRNAs linked to cell wall modification are differentially expressed (Gorshkova et al., 2018; Gorshkov et al., 2019). Modification of walls to form intercellular spaces is also observed in the development of leaf mesophyll cells that present a multipolar type of growth (Panteris and Galatis, 2005). These cells present a variety of shapes during their development, which is driven by microtubules and actin filaments. They go from being densely packed polyhedral cells without intercellular spaces, to large, branched and separated from each other by intercellular spaces with indentations and lobed regions (Panteris et al., 1993). The lobes are the regions with tip growth where microtubules are organized perpendicularly to the cell axis in the base of the lobes but are absent in the tip where additional actin filaments are abundant, similar to pollen tubes (Zhang et al., 2021).

In laticifer development, cell division in ground meristem originates a daughter cell through the formation of the cell plate and not by nuclear divisions. Then, the daughter cell differentiates into a laticiferous cell whose terminal walls may be observed in the laticifer apex (Milanez, 1960, 1977; Gama et al., 2017). We observed that in the apical region, intercellular spaces are absent between laticifer and neighboring cells, corroborating observations from other studies (Milanez, 1959; Demarco et al., 2006; Demarco and Castro, 2008; Medina et al., 2021). In particular, in the case of branched laticifers of *Asclepias*, where the laticifer cells can be a sinuous cylinder, they maintain the cell-to-cell wall adhesion with neighboring cells, contrary to the pattern observed in mesophyll cells, and the transverse arrangement of microtubules is uniform throughout the entire row of cells, not displaying differential growth or any different patterns that could promote polarized growth, as observed in unbranched laticifers. Sousa-Baena et al. (2021) observed cortical microtubules transversally arranged in hypocotyl epidermal cells in *Ipomoea nil*, a very similar pattern observed in cortical microtubules of both branched and unbranched laticifers in this study.

### Laticifers grow diffusely and display transversally oriented microtubules

Although laticifers are not essential for basic plant development (Castelblanque et al., 2016; Benninghaus et al., 2020), they are important in providing defense through the production of latex, which is, in fact, energetically expensive to produce in both primary and secondary metabolism (Agrawal and Konno, 2009). It is important to consider that during laticifer development and elongation, the latex production occurs simultaneously. Depending on the species, terminal walls are also being dissolved at the same time. It can represent a very high cost to the plant if, at the same time, modifications to the organization of cortical microtubules are required for polarized growth, as occur in pollen tubes. Li et al. (2017) describe this as an extreme polarized mode of plant cell growth that requires a high activity in the tip to penetrate the style and reach the ovule through intrusive growth. In pollen tubes, microtubules are organized in longitudinal bundles along the tube elongation axis and are absent in its apical and subapical regions (Cai et al., 2017). Conversely, we show that the cortical microtubules of laticifers are transversally arranged, following a pattern that is considered the default organization which allows the cell to organize the cytoskeleton during organ elongation (Wasteneys, 2004).

Laticifer terminal wall degradation and the branching could also be mediated by microtubules. In pit formation on vessel elements of *Aesculus hippocastanum*, cortical microtubules form rings around the perforation but not in the pit; instead, they are randomly arranged between the adjacent pits (Chaffey et al., 1997). A similar process was observed in pit formations of fibers in the hybrid *Populus tremula × P. tremuloides* (Chaffey et al., 2002). In protoxylem (Schneider et al., 2021) and metaxylem (Schneider et al., 2017) formation in *Arabidopsis*, microtubules form the template for cell wall deposition, and in areas where the cortical microtubules density is reduced, the cell wall is thinner. Canaveze et al. (2019) observed that meristematic cells in contact with laticifers, which subsequently may be incorporated into the laticifer system, presented microtubules perpendicularly oriented with respect to axis growth. However, more studies are necessary to better understand the role of microtubules in branched laticifers.

Our study showed that laticifers have a coordinated growth with the neighboring cells, in which the cortical microtubules are arranged in a perpendicular orientation to the cell axis. The same microtubule arrangement was observed in ground meristem, parenchyma and secretory idioblasts, indicating that all these cells have diffuse growth. No sign of polarized growth was observed in laticifers. This is the first study of microtubules in laticifers and living image technologies; transcriptomic studies of laticifers could be also a good strategy to investigate the developmental process and mode of growth of these complex secretory cells.

## Data availability statement

The original contributions presented in the study are included in the article/supplementary material, further inquiries can be directed to the corresponding authors.

## Author contributions

MM: conceptualization, data curation, formal analysis, investigation, methodology, and writing—original draft, and writing—review and editing. MS-B: data curation, formal analysis, methodology, and writing—review and editing. M-AS: data curation and writing—review and editing. DD: conceptualization, data curation, formal analysis, methodology, funding acquisition, project administration, supervision, writing—original draft, and writing—review and editing. All authors contributed to the article and approved the submitted version.

## Funding

This work was funded by CAPES—Coordenacṃão de Aperfeicṃoamento de Pessoal de Nível Superior (Grant number 001), Conselho Nacional de Desenvolvimento Científico e Tecnológico (CNPq proc. #304297/2021–6), and FAPESP—Fundacṃão de Amparo à Pesquisa do Estado de São Paulo (proc. #2017/23882–0 and #2022/15580-1)].

## Conflict of interest

The authors declare that the research was conducted in the absence of any commercial or financial relationships that could be construed as a potential conflict of interest.The reviewer ST declared a shared affiliation with the authors to the handling editor at the time of review.

## Publisher’s note

All claims expressed in this article are solely those of the authors and do not necessarily represent those of their affiliated organizations, or those of the publisher, the editors and the reviewers. Any product that may be evaluated in this article, or claim that may be made by its manufacturer, is not guaranteed or endorsed by the publisher.
