# Corrigendum: Laticifer growth pattern is guided by cytoskeleton organization

**DOI:** 10.3389/fpls.2023.1198197

**Published:** 2023-06-22

**Authors:** Maria Camila Medina, Mariane S. Sousa-Baena, Marie-Anne Van Sluys, Diego Demarco

**Affiliations:** Departamento de Botânica, Instituto de Biociências, Universidade de São Paulo, São Paulo, SP, Brazil

**Keywords:** laticifers, cytoskeleton, microtubules, development, apical growth

**Incorrect Funding**

In the published article, there was an error in the **Funding** statement, an incorrect number was used.

The previous Funding statement:

“This work was funded by CAPES—Coordenação de Aperfeiçoamento de Pessoal de Nível Superior (Grant number 001), Conselho Nacional de Desenvolvimento Científico e Tecnológico (CNPq proc. #304297/2021–6), and FAPESP— Fundação de Amparo à Pesquisa do Estado de São Paulo (proc. #2017/23882–0 and #2022/11286-1).”

The corrected Funding statement appears below.

This work was funded by CAPES—Coordenação de Aperfeiçoamento de Pessoal de Nível Superior (Grant number 001), Conselho Nacional de Desenvolvimento Científico e Tecnológico (CNPq proc. #304297/2021–6), and FAPESP—Fundação de Amparo à Pesquisa do Estado de São Paulo (proc. #2017/23882–0 and #2022/15580-1).

The authors apologize for this error and state that this does not change the scientific conclusions of the article in any way. The original article has been updated.

